# Electrical impedance measurements can identify red blood cell–rich content in acute ischemic stroke clots *ex vivo* associated with first-pass successful recanalization

**DOI:** 10.1016/j.rpth.2024.102373

**Published:** 2024-03-15

**Authors:** Cansu Sahin, Alice Giraud, Duaa Jabrah, Smita Patil, Pierluca Messina, Franz Bozsak, Jean Darcourt, Federico Sacchetti, Anne-Christine Januel, Guillaume Bellanger, Jorge Pagola, Jesus Juega, Hirotoshi Imamura, Tsuyoshi Ohta, Laurent Spelle, Vanessa Chalumeau, Uros Mircic, Predrag Stanarčević, Ivan Vukašinović, Marc Ribo, Nobuyuki Sakai, Christophe Cognard, Karen Doyle

**Affiliations:** 1 Department of Physiology, University of Galway, Galway, Ireland; 2 Centre for Research in Medical Devices (CÚRAM)- Science Foundation Ireland (SFI), University of Galway, Galway, Ireland; 3 Sensome, Massy, France; 4 Department of Diagnostic and Therapeutic Neuroradiology, Centre Hospitalier Universitaire (CHU) de Toulouse, Toulouse, France; 5 Department of Neurology, University Hospital Vall d’Hebron, Barcelona, Spain; 6 Department of Neurosurgery, Kobe City Medical Center General Hospital, Kobe, Japan; 7 Department of Interventional Neuroradiology, Bicêtre Hospital, Le Kremlin-Bicêtre, France; 8 Department of Neuroradiology, Centre for Radiology and Magnetic Resonance Imaging (MRI), University Clinical Center of Serbia, Belgrade, Serbia; 9 Neurology Clinic, University Clinical Center of Serbia, Belgrade, Serbia

**Keywords:** acute ischemic stroke, clot composition, electrical impedance, first-pass effect, mechanical thrombectomy

## Abstract

**Background:**

Electrochemical impedance spectroscopy can determine characteristics such as cell density, size, and shape. The development of an electrical impedance–based medical device to estimate acute ischemic stroke (AIS) clot characteristics could improve stroke patient outcomes by informing clinical decision making.

**Objectives:**

To assess how well electrical impedance combined with machine learning identified red blood cell (RBC)–rich composition of AIS clots *ex vivo*, which is associated with a successfully modified first-pass effect.

**Methods:**

A total of 253 clots from 231 patients who underwent thrombectomy in 5 hospitals in France, Japan, Serbia, and Spain between February 2021 and October 2023 were analyzed in the Clotbase International Registry. Electrical impedance measurements were taken following clot retrieval by thrombectomy, followed by Martius Scarlet Blue staining. The clot components were quantified via Orbit Image Analysis, and RBC percentages were correlated with the RBC estimations made by the electrical impedance machine learning model.

**Results:**

Quantification by Martius Scarlet Blue staining identified RBCs as the major component in clots (RBCs, 37.6%; white blood cells, 5.7%; fibrin, 25.5%; platelets/other, 30.3%; and collagen, 1%). The impedance-based RBC estimation correlated well with the RBC content determined by histology, with a slope of 0.9 and Spearman’s correlation of r = 0.7. Clots removed in 1 pass were significantly richer in RBCs and clots with successful recanalization in 1 pass (modified first-pass effect) were richer in RBCs as assessed using histology and impedance signature.

**Conclusion:**

Electrical impedance estimations of RBC content in AIS clots are consistent with histologic findings and may have potential for clinically relevant parameters.

## Essentials

• An electrical impedance–based medical device may be useful to estimate stroke clot characteristics.
• Red blood cell (RBC) content was assessed by histology and estimated using electrical impedance.
• The impedance-based RBC estimation correlated well with the RBC content determined by histology.
• Clots removed in 1 pass and with successful recanalization in the first pass are richer in RBCs.

## 1 Introduction

Acute ischemic stroke (AIS) accounts for 87% of all stroke cases [1]. Thrombi can be removed from cerebral vessels using thrombolytic therapy (recombinant tissue plasminogen activator [rtPA]) and/or by mechanical thrombectomy [2], an interventional image-guided procedure by which a thrombus is removed using an endovascular device [3].

Mechanical thrombectomy is very effective in the treatment of large intracranial vessel occlusion [4]. First-pass effect, defined as achieving complete recanalization with a single thrombectomy pass, is strongly correlated with better functional outcomes and lower mortality rates [5]. Modified first-pass effect (mFPE), defined as achieving successful recanalization of modified thrombolysis in the cerebral infarction (mTICI) 2b or better after the first pass, is also associated with better patient outcomes [5,6].

Thrombi are primarily composed of red blood cells (RBCs), white blood cells (WBCs), fibrin, platelets/other, and collagen [7]. However, it is known that there is marked heterogeneity in clot composition [8]. The effects of thrombus characteristics on clinical outcomes after AIS have been investigated by several groups [9,10]. Several large studies have shown that fibrin and platelet-rich clots are more difficult to remove than RBC-rich clots [[11], [12], [13]].

A hyperdense artery sign on computed tomography and a positive susceptibility sign on magnetic resonance imaging can indicate a thrombus that is rich in RBCs, but accurate detection of thrombus characteristics with diagnostic scans remains challenging at present [14,15]. Development of a technology to reliably estimate thrombus composition in the acute care setting would be a great advance.

Electrical properties of cells and tissues can provide an insight into their structure and composition, depending on the frequency of the electrical signal applied. Application of a sinusoidal current to tissue between 2 electrodes and subsequent measurement of the current response gives a characteristic electrical impedance spectrum over a range of frequencies [16]. At low frequencies, cells act as insulators, so the current passes around them, and the electrical impedance signature is sensitive to cell size and density. At higher frequencies, due to the high capacitance of the cell membrane, the current passes through the cells, and the electrical impedance signature is sensitive to the cell type [17,18].

Combining electrical impedance spectra with machine learning algorithms to estimate tissue composition may be useful to characterize thrombus composition in the stroke acute care setting, providing neurointerventionalists with valuable information to inform therapy decisions [[19], [20], [21]].

We previously published pilot data that showed the potential of electrical impedance to recognize RBC components in AIS clots in the Clotbase Pilot Study [22]. One aim of this study was to evaluate how well electrical impedance analysis could identify the RBC composition of 253 clots collected in 231 cases from the Clotbase International Registry of AIS clots, confirming the pilot study observations. Furthermore, using RBC content as an indicator, we investigated the association of the electrical impedance signature with clots that were removed in 1 pass from those requiring more than 1 pass and association with successful mFPE.

## 2 Methods

### 2.1 Patient cohort and baseline characteristics

**Figure 1:**
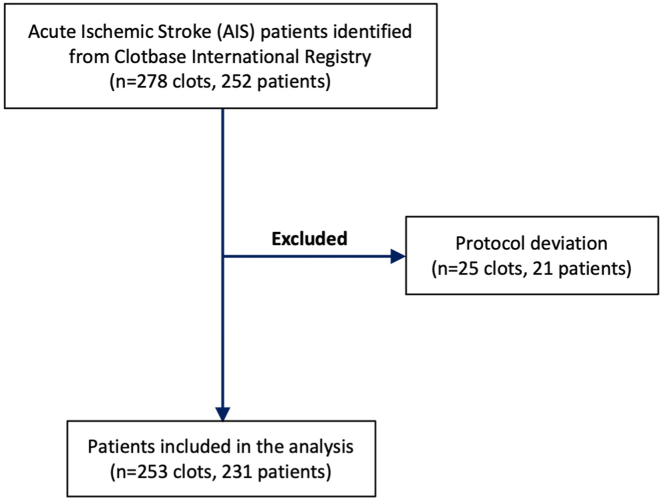
Patient selection flowchart. AIS, acute ischemic stroke.

In this study, a total of 253 clots from 231 patients were analyzed as part of the Clotbase International Registry of AIS clots and histologic analysis with electrical impedance data. Ethical approval for the study was obtained from the hospital research ethics committees of the 5 hospitals involved in the study: Purpan Hospital, Toulouse, France; Kobe City Medical Center General Hospital, Kobe, Japan; Vall d’Hebron Hospital, Barcelona, Spain; University Clinical Center of Serbia, Belgrade, Serbia; Bicêtre Hospital, Paris, France; and the University of Galway Research Ethics Committee (19-OCT-08). Patients were included if they were aged 18 years or older, with a large vessel occlusion causing ischemic stroke, had undergone mechanical thrombectomy treatment for AIS between the dates of February 4, 2021, and October 3, 2023, in one of the participating stroke centers, and for whom successful electrical impedance signature of the extracted thrombus was obtained prior to histologic analysis of the clot. Patient information including age, sex (female or male), thrombolysis treatment, occlusion location, suspected etiology, stroke severity (National Institutes of Health Stroke Scale/Score [NIHSS] on admission and at discharge), recanalization success (mTICI), and stroke risk factors was collected. Exclusion criteria were protocol deviations that occurred, such as resulting from an electrical issue during the impedance measurement process. In total, 231 AIS patients were identified for the analysis (Figure 1).

**Figure 2:**
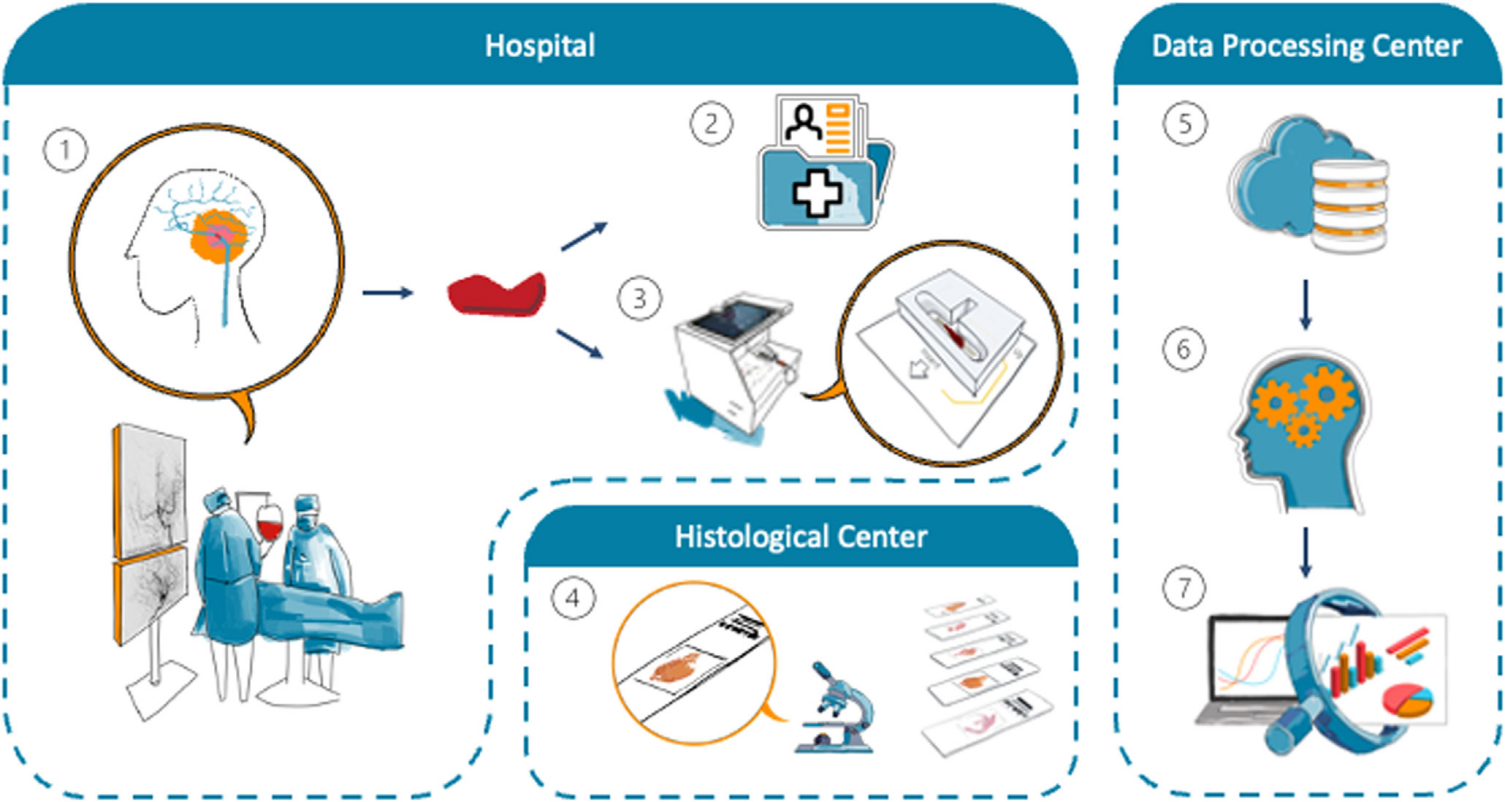
Workflow: mechanical thrombectomy (1), patient information (2), electrical impedance measurements (3), histology (4), cloud database (5), machine learning (6), and correlation analysis (7).

Figure 2 shows the overall procedural workflow of this study, which included mechanical thrombectomy as treatment for AIS, clot retrieval, *ex vivo* electrical impedance measurements, processing of clot samples for histologic analysis, and correlation of impedance and histologic data with patient information.

### 2.2 Electrochemical impedance spectroscopy

Following mechanical thrombectomy, the thrombus was placed in a single-use cartridge and kept moist with application of 750 μL of phosphate-buffered saline, and a gross photograph was taken before electrochemical impedance spectroscopy (EIS) analysis. Each custom-made single-use cartridge contained an array of 48 gold electrodes spaced 450 μm apart that facilitated 45 individual impedance measurements between adjacent electrodes as previously described [22]. Care was taken to ensure contact between the thrombus tissue and electrode array. A high-precision electrical impedance analyzer (ISX-3, Sciospec GmbH) was used to measure the electrical impedance signature in the frequency range between 1 kHz and 30 MHz.

The phosphate-buffered saline signal from the electrode pairs not in contact with the thrombus was isolated from the thrombus signal. Following this step, the average number of individual measurements per thrombus was found to be 20.1 ± 10.5. These individual measurements were further processed in order to eliminate electrical parasitic effects. The machine learning algorithm was trained on an impedance data training set to differentiate thrombus components. In order to recognize the RBC signature for each individual measurement, a support vector machine classifier was trained, as previously described [22]. We employed a 5-fold cross-validation approach to assess the efficacy of the model and maximize data utilization [23]. By dividing into 5 distinct subsets, cross-validation allows multiple iterations of model training and testing. This guarantees that each thrombus in the training set functions as both a training and test sample (additional detail in Supplementary Information). The data collection is still ongoing to create a large validation set that will be used to develop a confirmatory predictive model (as recommended by Transparent Reporting of a multivariable prediction model for Individual Prognosis Or Diagnosis guidelines).

### 2.3 Histopathologic compositional analysis

Following the impedance measurements, the samples were immediately placed in 10% neutral buffered formalin and sent to the University of Galway for histologic analysis. All clots were then processed using a fully enclosed tissue processor (Leica, ASP300s). Three-micrometer sections were taken from paraffin-embedded clots and stained with Martius Scarlett Blue, which allows differentiation of the main clot components: RBCs, WBCs, fibrin, platelets/other, and collagen [24,25].

### 2.4 Quantification methods

Stained slides were scanned using the Olympus VS120 Virtual Slide Scanner with a 20× objective. Clot components were classified and quantified using Orbit Image Analysis software (www.orbit.bio), which is an open-source whole slide image analysis tool, as previously described [26,27]. The quantification model was trained to discriminate between clot components and background based on their color.

### 2.5 Statistical analysis

Normality of data was assessed using the Kolmogorov–Smirnov test. As the data were not normal, to compare the 2 groups, Mann–Whitney U-test was used. For comparisons across multiple groups, the Kruskal–Wallis nonparametric test followed by Dunn’s multiple comparisons test were used. For correlation analysis, the nonparametric Spearman’s correlation coefficient was calculated using a simple linear regression model. All statistical tests were 2-sided, and the results were regarded as statistically significant if the *P* value was less than .05. GraphPad Prism 9 (GraphPad Software, La Jolla, CA) was used for analysis.

## 3 Results

### 3.1 Percentage of main clot components and clot heterogeneity as assessed by histology

The mean percentage (±SD) of the main components in the samples was as follows: RBCs, 37.6% (±21.7%); WBCs, 5.7% (±3.9%); fibrin, 25.5% (±14.9%); platelets/other 30.3% (±17.5%); and collagen 1% (±7.2%). As expected and in line with previously published findings, there was marked heterogeneity in main components across the clot population. RBC percentage composition ranged from 95.6% in the most RBC-rich thrombus to 0.03% in the least RBC-rich thrombus. Supplementary Figure S1 shows an image of a representative Martius Scarlett Blue–stained clot.

### 3.2 Baseline characteristics and associated clot composition

Baseline characteristics are presented in Table 1. The median age of this cohort was 76 (IQR, 84-65) years (*N* = 229). Data by age groups (<50, 50-59, 60-69, 70-79, 80-89, and ≥90 years) [28] are reported in Table 1. While there was no significant difference between the age groups in terms of RBC, WBC, and platelets/other contents, there was significantly more fibrin in the clots of the older age groups (Supplementary Table S1).

**Table 1:** Baseline characteristics of patients.

Characteristic	Samples	
Age	*n*	%
<50 y	16	7.0
50-59 y	17	7.4
60-69 y	40	17.5
70-79 y	69	30.1
80-89 y	69	30.1
≥90 y	18	7.9
Total	229	100
Sex		
Female	112	50.0
Male	112	50.0
Total	224	100
Recombinant tissue plasminogen activator		
Yes	148	64.1
No	83	35.9
Total	231	100
Occlusion locations		
M1	126	49.8
Intracranial ICA/terminus	44	17.4
M2	27	10.7
Basilar	14	5.5
Cervical ICA	9	3.6
Vertebral	3	1.2
P2	1	0.4
Multiple	29	11.5
Total	253	100
Suspected etiology		
Large-artery atherosclerosis	31	13.5
Cardioembolism	156	67.8
Cryptogenic	22	9.6
Other	21	9.1
Total	230	100
NIHSS on admission		
Mild stroke <6	10	4.3
Moderate stroke 6-15	75	32.5
Severe stroke >15	146	63.2
Total	231	100
NIHSS at discharge		
Mild stroke <6	113	52.3
Moderate stroke 6-15	60	27.8
Severe stroke >15	43	19.9
Total	216	100
Modified thrombolysis in cerebral infarction score		
0	2	0.9
1	2	0.9
2a	3	1.3
2b	36	15.6
2c	56	24.2
3	132	57.1
Total	231	100

ICA, internal carotid artery; NIHSS, National Institutes of Health Stroke Scale/Score.

Overall, 50% of patients were female (Table 1). No significant differences were found between sexes in main clot components, with the exception of collagen, which was significantly higher in females (Supplementary Table S1).

Sixty-four percent of patients received rtPA (Table 1). In line with previous reports in the literature [29,30], rtPA administration did not significantly alter the average percentage of any clot component (Supplementary Table S1).

The most common occlusion location was the M1 segment of the middle cerebral artery (Table 1). No statistical difference in clot composition in clots extracted from different occlusion locations was observed (Supplementary Table S1). Suspected etiology was classified into 5 stroke subtypes according to the Trial of ORG 10172 in Acute Stroke Treatment, or TOAST, classification [31]. Most strokes were suspected cardioembolic in origin (Table 1). Although there was no overall statistically significant difference in composition, RBC composition was highest in large artery atherosclerosis etiology, in line with previously published findings [12] (Supplementary Table S1).

NIHSS scores were grouped according to severity of stroke [32] as follows: <6, mild stroke; 6 to 15, moderate stroke; and >15, severe stroke (Table 1). There was no significant difference in clot composition observed according to NIHSS at admission (Supplementary Table S1). The NIHSS score at discharge was available in 216 patients (93.5%; Table 1). There was also no significant difference in clot composition observed according to NIHSS at discharge (Supplementary Table S1). Recanalization success was graded with the mTICI score system [33]. Successful recanalization was not achieved in 7 patients (3.1%). Successful recanalization was achieved for the remaining 224 patients (Table 1). mTICI was not significantly associated with differences in the percentage of clot components (Supplementary Table S1).

Overall, 28% (*n* = 65) of patients had a single risk factor. Among patients with 1 risk factor, hypertension was the most prevalent factor (24.3%), and atrial fibrillation was the second most common (18.9%). However, most patients (68%, *n* = 157) had multiple risk factors. Two risk factors were the most common number of risk factors in the patient cohort. Of patients with 2 or more risk factors, the most common risk was hypertension (26.5%), followed by atrial fibrillation (18.7%) and hyperlipidemia (17.8%). There was no statistically significant difference in clot composition associated with stroke risk factors (Supplementary Table S2).

### 3.3 Per-pass histologic analysis

A total of 166 clots were retrieved in 1 pass only, whereas 87 clots required more than 1 pass (Table 2). The analysis of thrombus components per pass showed that RBC percentage in clots retrieved in a single pass was significantly higher than that in clots that required more than 1 pass for retrieval (Table 2). In line with this, the percentage of fibrin was significantly higher in clots that required more than 1 pass to retrieve (Table 2). There was no significant difference between thrombus fragments retrieved in 1 pass or requiring more than 1 pass with regard to WBCs, platelets/other, and collagen (Table 2).

**Table 2:** Histologic assessment of main clot components analyzed per pass and according to successful recanalization outcome (modified first-pass effect).

Revascularization outcome	Samples		Histologic composition (MSB), mean ± SD				
Per-pass analysis	*n*	%	RBC	WBC	Fibrin	Platelets/other	Collagen
1 pass	166	65.6	41.1 ± 22.4	5.2 ± 3.6	23.5 ± 14.3	29.3 ± 17.1	0.9 ± 7.5
>1 pass	87	34.4	33.3 ± 22.1	6.2 ± 4.5	27.9 ± 16.2	31.8 ± 18.9	0.8 ± 5.6
Total	253	100	U = 5691; *P* = .006^a^	U = 6348; *P* = .1	U = 6023; *P* = .03^b^	U = 6729; *P* = .4	U = 6706; *P* = .4

mFPE	*n*	%	RBC	WBC	Fibrin	Platelets/other	Collagen
mFPE	152	65.8	40.4 ± 21.4	5.3 ± 3.5	24.0 ± 13.7	29.3 ± 16.8	1.0 ± 7.8
non-mFPE	79	34.2	32.4 ± 21.6	6.3 ± 4.6	28.3 ± 16.8	32.2 ± 18.8	0.9 ± 5.8
Total	231	100	U = 4628; *P* = .004*a*	U = 5431; *P* = .2	U = 5143; *P* = .07	U = 5553; *P* = .4	U = 5848; *P* = .7

mFPE, modified first-pass effect; MSB, Martius Scarlett Blue; RBC, red blood cell; WBC, white blood cell.

a P ≤ .01.

b *P* ≤ .05; Mann-Whitney U test.

### 3.4 mFPE assessed by histology

mFPE was analyzed per case. Unsuccessful recanalization (mTICI 0-2a) was reported in 7 cases. mFPE (1 pass, mTICI 2b or better) was achieved for 152 of the cases. mFPE was not achieved in 79 cases (non-mFPE group, including both mTICI 0-2a [*n* = 7] and >1 pass [*n* = 72]). The RBC percentage was significantly higher in the mFPE group than in the non-mFPE group (Table 2). There were no other statistically significant differences observed for other components: WBCs, fibrin, platelets/other, and collagen (Table 2).

### 3.5 Correlation between RBC percentage composition and electrical impedance signature

**Figure 3:**
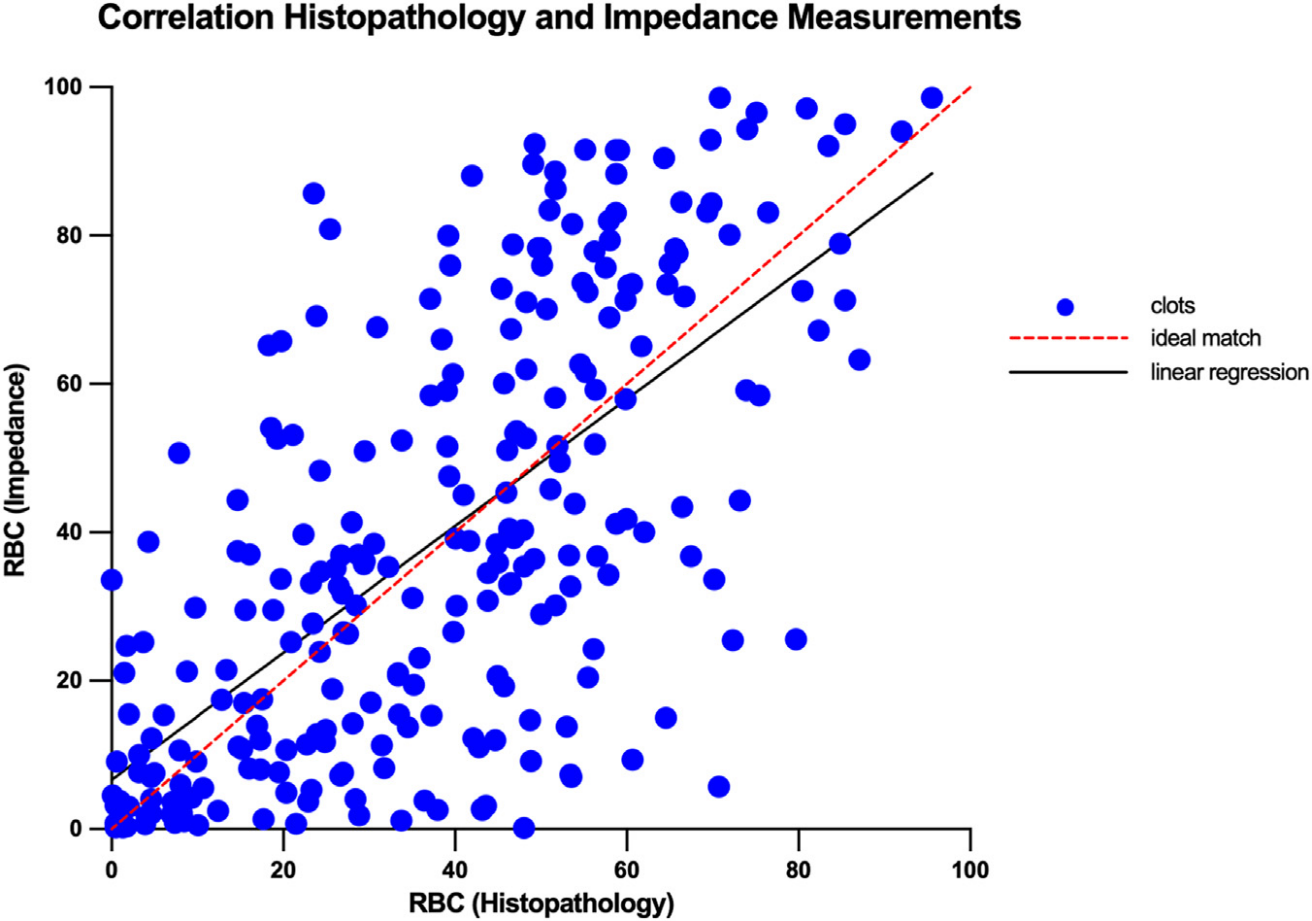
Correlation of red blood cell (RBC) percentage composition as measured by histology and estimated from impedance data using simple linear regression. Equation of the line: r_m_ = 0.9 r_h_ + 7, *N* = 253.

There was a strong relationship between RBC percentage composition assessed by histology and the electrical impedance signature–based RBC composition (Figure 3). Linear regression analysis indicated a slope of 0.9 with a 7% deviation from the origin (r_m_ = 0.9, r_h_ + 7). The impedance-based RBC algorithm output was positively correlated with the RBC content determined by histology, with a significant Spearman’s correlation of r = 0.7 and ∗∗∗*P* < .001.

To further interrogate the difference between the 2 measurements, we carried out Bland–Altman analysis [34] to assess the limits of agreement. Bland–Altman analysis revealed a mean bias (average of differences) of 1.1% between histopathology and estimated impedance measurements. Ninety-five percent CI limits of agreement ranged from −42.4% to 44.5% (Supplementary Figure S2).

### 3.6 Association of electrical impedance RBC signature with clots removed in 1 pass and requiring more than 1 pass

**Figure 4:**
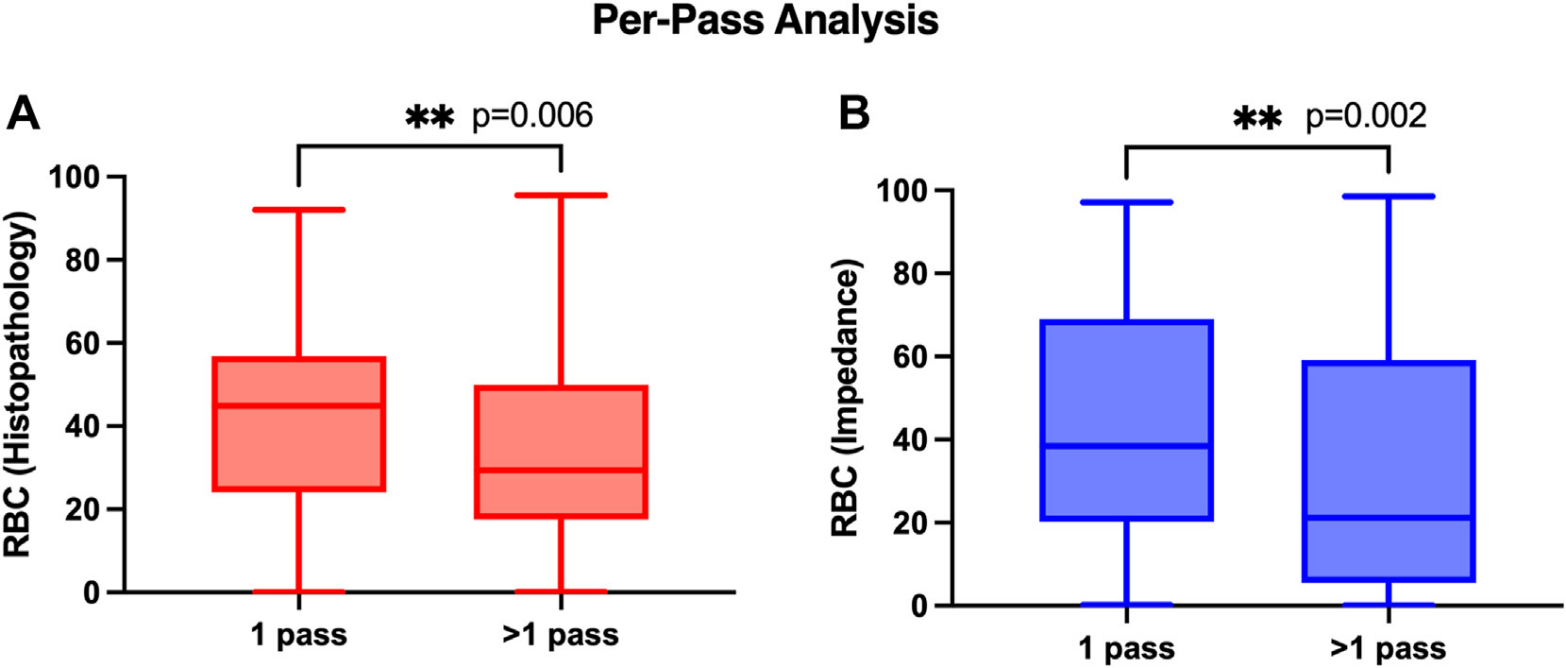
Per-pass analysis of (A) red blood cell (RBC) percentage by histology and (B) RBC composition based on impedance measurements. ∗∗*P* ≤ .01.

As previously shown in Table 2, the RBC percentage of thrombi retrieved in 1 pass (41.1%) was significantly higher than those retrieved in >1 pass (33.3%) when analyzed histologically (Figure 4A). Analysis of the impedance data using a machine learning model to identify RBC percentage also indicated the same significant effect (1-pass RBC percentage, 43.2 ± 27.8; >1-pass RBC percentage, 32.5 ± 30.7; *n* = 253; U = 5469; *P* = .002; Figure 4B).

To make a direct comparison of the impedance-derived data on clots retrieved from the same occlusion location, we analyzed the subset of samples extracted from the most common occlusion site, the M1 segment of the middle cerebral artery. One hundred twenty-six (49.8%) of the clots collected in this study were extracted from the M1. A similar profile of the effect of the pass was observed in clots removed in this M1 subset as in the main analysis (histologic assessment: 1-pass [*n* = 92] RBC percentage, 39.9 ± 21.4; >1-pass [*n* = 34] RBC percentage, 27.6 ± 22.1; *n* = 126; U = 998; *P* = .002) and RBC percentage from impedance measurements (1 pass RBC percentage, 43.0 ± 27.0; >1 pass RBC percentage, 24.6 ± 25.1; *n* = 126; U = 901; *P* = .0002).

### 3.7 Association of electrical impedance RBC signature with mFPE

**Figure 5:**
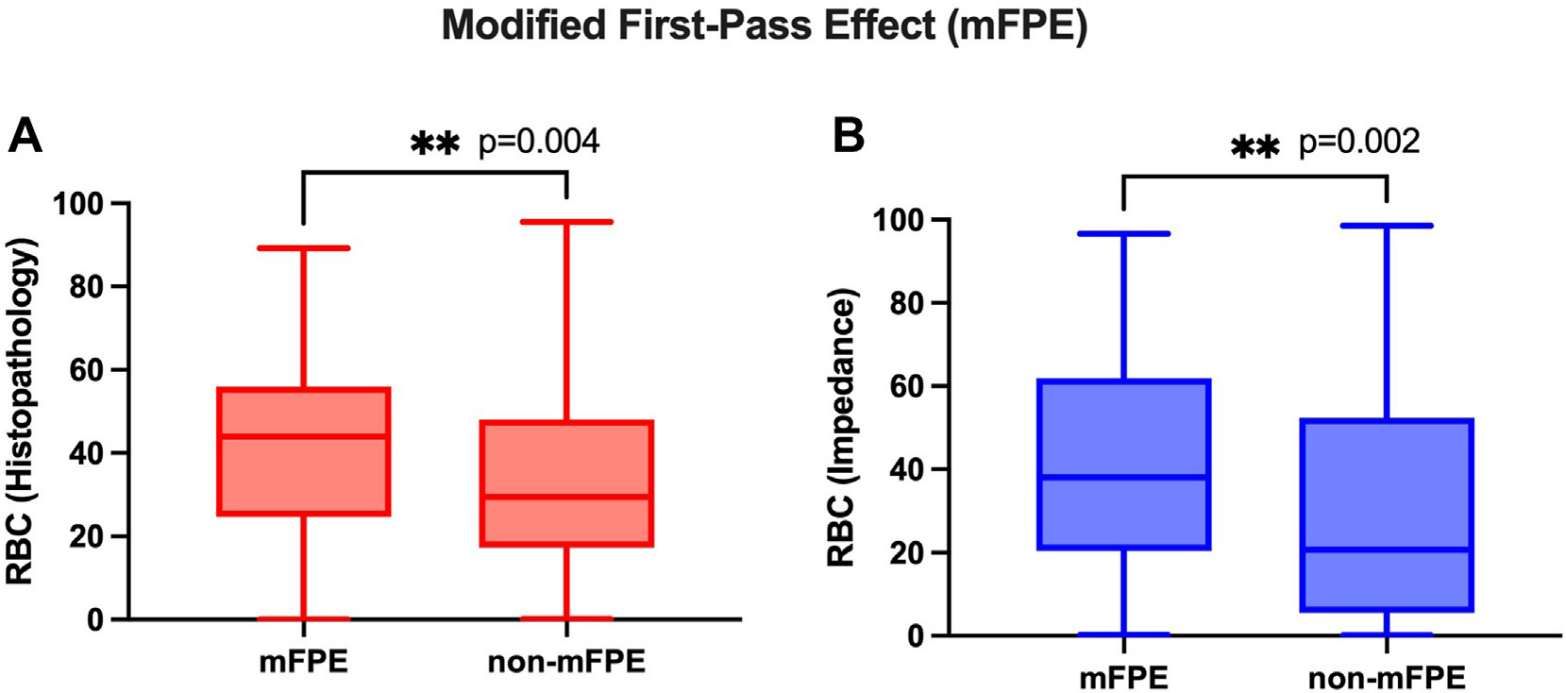
Modified first-past effect (mFPE) analysis of (A) red blood cell (RBC) percentage by histology and (B) RBC percentage based on impedance measurements.

The clots in the mFPE group had significantly higher RBC composition by histologic analysis (40.4% vs 32.4%; Figure 5A), and this was also apparent with the RBC estimation using impedance measurements (mFPE RBC percentage, 42.6 ± 26.8; non-mFPE RBC percentage, 32.0 ± 29.7; *n* =231; U = 4495; *P* = .002; Figure 5B).

## 4 Discussion

Understanding the histologic characteristics of AIS thrombi and studying the relationship between thrombus characteristics and clinical factors in stroke patients can provide insight into stroke pathophysiology and inform developments in medical device design and acute stroke treatment approach. Clot composition is a determinant that may affect the success of thrombolysis [35] and mechanical thrombectomy [11,12,36]. RBC content and the extent of the whiter fibrin and platelet components have been identified as key features. Clots rich in platelets and fibrin require more passes for extraction [12]. Recently published findings have shown that electrical impedance measurements can be used to train a machine learning algorithm to successfully identify the RBC-rich composition of a thrombus *ex vivo* [16]. Using a larger sample size, the current study builds on this and shows for the first time that EIS can be positively associated with clinically relevant parameters such as likelihood of retrieval in the first pass and successful recanalization in the first pass.

As expected, there was a strong correlation between observed RBC percentage by histology and RBC percentage using electrical impedance signatures. Histologic analysis indicated that the thrombus removed in the first pass was significantly richer in RBCs than the thrombus that required more than 1 pass to remove, in line with previously published findings [11,12,36]. RBC percentage estimated using the electrical impedance signature was significantly different in the clots that were removed in 1 pass from the more difficult-to-remove clots. Furthermore, histologic analysis showed that thrombi removed in procedures with successful recanalization in the first pass were richer in RBCs than those in which mFPE was not achieved, in line with observations in the literature [[37], [38], [39], [40]]. RBC percentage estimated using the electrical impedance signature was also significantly different in the clots removed with successful recanalization, suggesting that the electrical impedance analysis may have potential as a useful diagnostic tool to inform the clinician of thrombus characteristics in the acute care setting.

The patient baseline characteristics demonstrate that almost two-thirds of patients were administered thrombolysis before thrombectomy. Similar to previously published observations, we found that there was no difference in the proportion of clot components with thrombolysis [7,30]. The most common occlusion location was the M1 segment of the middle cerebral artery. No difference in clot composition according to vessel occlusion location was observed. The majority of strokes were suspected to be of cardioembolic origin; no significant difference in clot composition was observed, although there was a clear trend of higher RBC content in large-artery atherosclerotic etiology, in line with 2 of the largest studies published to date [12,30]. There is no consensus in the literature regarding the relationship between stroke patient outcome and clot composition [41]. We found no significant difference in clot composition according to stroke severity on admission or discharge. Most patients had more than 1 risk factor, and hypertension was the most common risk factor identified, in line with a previously published report [42]. We observed no statistical difference in clot composition associated with stroke risk factors; however, further investigation of risk factors utilizing a larger sample size is necessary. It would also be interesting to explore the similarities and differences in extracted clots in recurrent stroke cases.

This study has several limitations. EIS analysis was conducted on thrombi *ex vivo* following removal by thrombectomy. EIS measurements are dependent on the electrodes coming into secure contact with the outer surface of the tissue, which can be challenging and possibly adversely affect tissue quality. Despite the presence of a cap in the hardware designed to press the clot onto the electrodes and the narrow confines of the well containing the thrombus, the control over the quality of this contact is not absolute and could lead to discrepancies in the RBC percentages observed in individual histologic and impedance measurements. Every effort was made to prevent the clot from drying out in air by keeping the thrombus in a physiological solution prior to impedance measurement, after which the tissue was fixed in formalin. A further limitation is that the impedance signature of the thrombus is taken from the clot surface in contact with the electrodes, while the histologic assessment of the clot arises from a transverse section from a midregion of the clot. While it is accepted that a single histologic slide can provide a qualitative representation of the entire thrombus, there can be considerable heterogeneity within a clot [43]. This variability could contribute to discrepancies in individual clot histologic and impedance measurements. Nonetheless, the very strong correlation between histologic and impedance population-based RBC composition suggests that the impedance reading may be capable of true estimation of RBC composition in individual cases with further development. There are also some limitations with the baseline characteristics of patient data, such as reported etiology is suspected but not confirmed at this time. We do not have information on race/ethnicity of patients, which would be valuable to explore further. We acknowledge that factors such as missing data from, for example, patient baseline characteristics could bias the results presented; however, the extent of missing data is low. Further work is ongoing to confirm baseline characteristics and increase sample number.

## 5 Conclusion

Electrical impedance estimations of RBC content in AIS clots are consistent with histologic findings and have potential for clinically relevant parameters. Thrombus removed in the first pass is richer in RBCs than clots that require more than 1 pass to remove, and clots removed with successful recanalization (mFPE) are also richer in RBCs. We found for the first time that the electrical impedance signature of RBCs may be associated with clinically relevant parameters such as likelihood of clot retrieval in the first pass and successful recanalization.
