# Supplementary figures and images for: Electrical impedance measurements can identify red blood cell–rich content in acute ischemic stroke clots ex vivo associated with first-pass successful recanalization
Source: Res Pract Thromb Haemost. 2024 Mar 15;8(3):102373. doi: 10.1016/j.rpth.2024.102373 (PMC11015511; doi:10.1016/j.rpth.2024.102373)

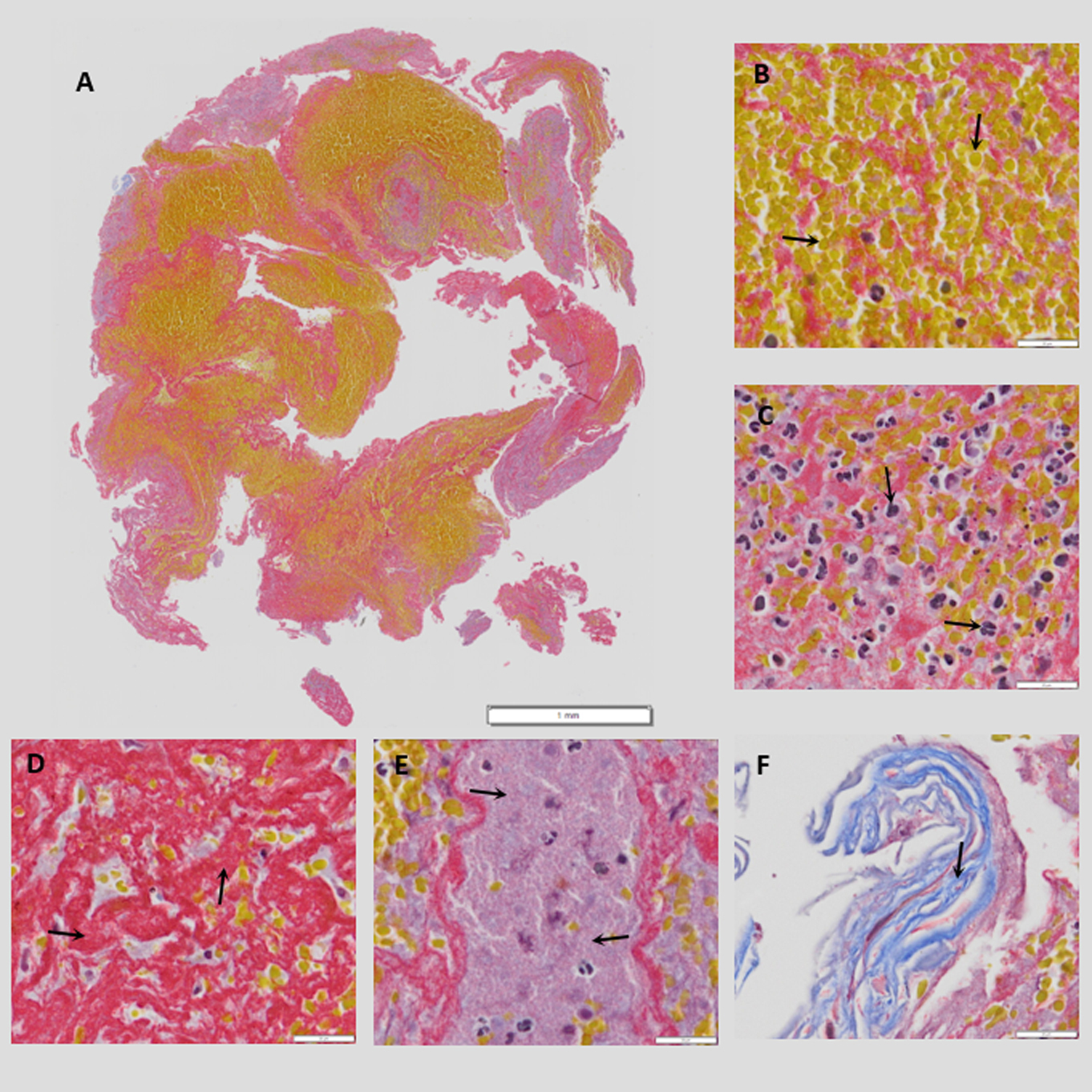

Supplement: Supplementary Figure 1 [file figs1.jpg]

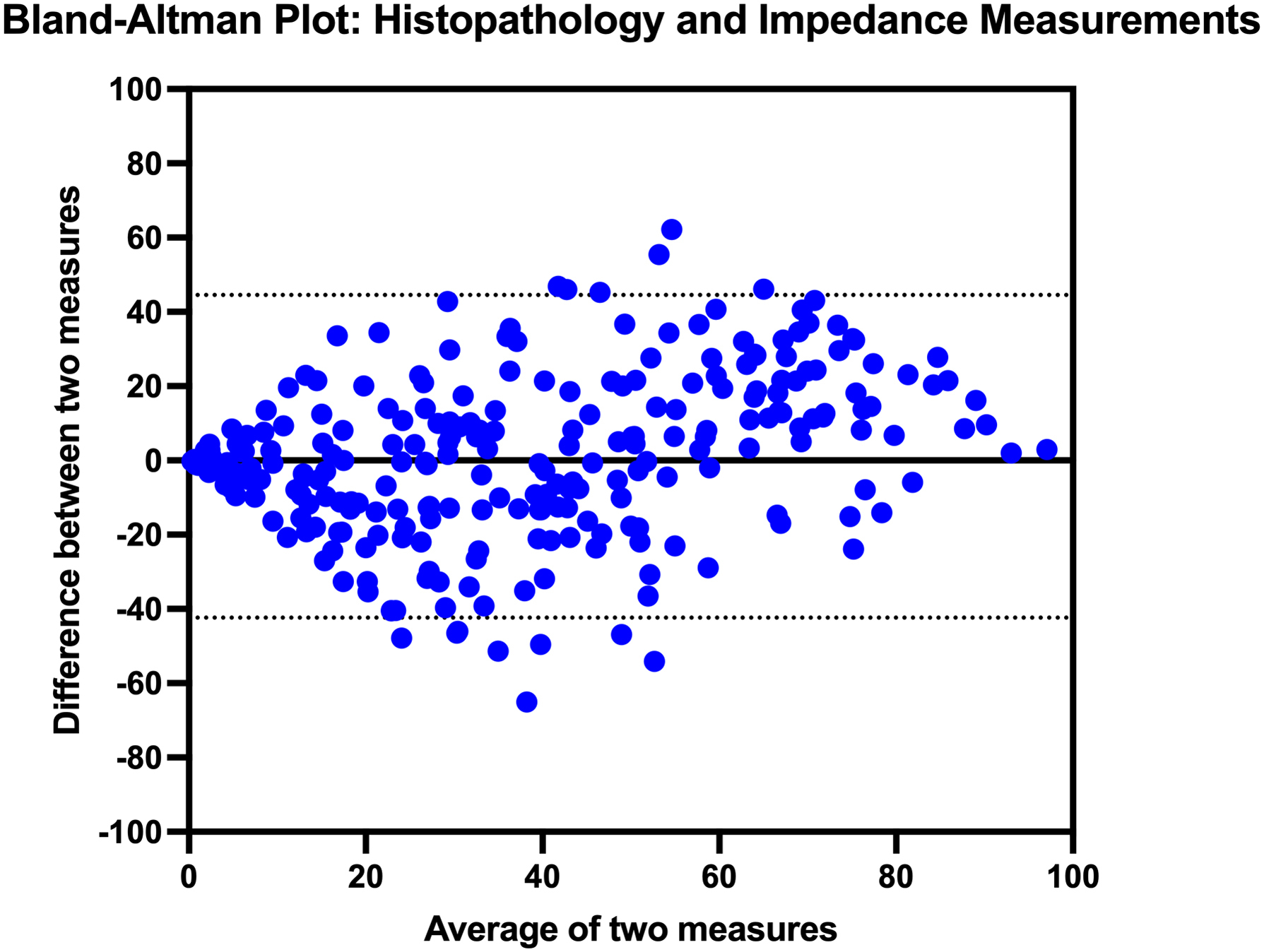

Supplement: Supplementary Figure 2 [file figs2.jpg]
