# Supplementary material for: Electrical impedance measurements can identify red blood cell–rich content in acute ischemic stroke clots ex vivo associated with first-pass successful recanalization
Source: Res Pract Thromb Haemost. 2024 Mar 15;8(3):102373. doi: 10.1016/j.rpth.2024.102373 (PMC11015511; doi:10.1016/j.rpth.2024.102373)

**Supplementary Information:** Support vector machine (SVM) classification method

A support vector classifier (SVC; also previously used in our in vitro work) was trained at the level of individual measurements to recognize the RBC signal of a given measurement. 29 thrombi were selected to train the model by one EIS expert (“training set”). Of these 29 thrombi, 10 had a RBC content above 70% based on histological analysis and 19 had a RBC content of less than 30% based on histological analysis. One EIS expert (AG 5 Years of expertise) validated that the 10 thrombi with high RBC content had individual measurements characteristic of RBC and that the 19 thrombi with low RBC content had individual measurements characteristic of absence of RBC. This selection ensured a balanced representation of individual measurements within each group, resulting in 319 and 396 measurements for thrombi with high and low RBC content, respectively. All individual measurements of the 10 thrombi with high RBC content were labelled as “RBC signal” and the measurements of the 19 thrombi with low RBC content were labelled as “not-RBC signal.” The output of the SVC is the probability of an individual measurement to be an RBC signal. To generate a singular estimation for a given thrombus, we aggregated the estimations for all individual measurements within that thrombus. This was achieved by averaging the probabilities of RBC signal presence across the individual measurements composing the thrombus. Consequently, the anticipated RBC content percentage for a thrombus corresponded to the resulting averaged probability.

**Supplementary Table 1.** Clot composition data associated with baseline characteristics

| **CHARACTERISTIC** | **HISTOLOGICAL COMPOSITION (MSB) Mean ± SD** | | | | |
| --- | --- | --- | --- | --- | --- |
| **Age** | **RBC** | **WBC** | **Fibrin** | **Platelets/other** | **Collagen** |
| <50 | 39.4 ± 22.0 | 5.6 ± 4.0 | 27.2 ± 15.8 | 27.2 ± 11.6 | 0.1 ± 0.3 |
| 50 - 59 | 36.8 ± 21.6 | 5.1 ± 4.1 | 20.1 ± 16.5^a^ | 37.8 ± 26.1 | 0.3 ± 0.7 |
| 60 - 69 | 39.6 ± 23.6 | 4.4 ± 3.1 | 21.3 ± 12.4^a^ | 34.2 ± 19.2 | 0.4 ± 1.3 |
| 70 - 79 | 42.0 ± 22.7 | 5.6 ± 3.6 | 23.2 ± 15.8^a^ | 29.2 ± 18.2 | 0.1 ± 0.2 |
| 80 - 89 | 33.3 ± 19.3 | 6.2 ± 4.3 | 27.8 ± 12.7 | 29.9 ± 14.9 | 2.7 ± 13.0 |
| ≥90 years | 32.6 ± 21.8 | 6.7 ± 4.7 | 35.9 ± 16.7 | 24.7 ± 13.4 | 0.2 ± 0.5 |
|  | **H(5)=6.8, p=0.2** | **H(5)=7.9, p=0.2** | **H(5)=20.4, p=0.001**** | **H(5)=5.8, p=0.3** | **H(5)=4.7, p=0.5** |
| **Sex** |  |  |  |  |  |
| Female | 35.4 ± 22.1 | 5.8 ± 3.9 | 26.5 ± 15.7 | 30.9 ± 18.4 | 1.4 ± 9.9 |
| Male | 40.1 ± 20.9 | 5.5 ± 4.0 | 24.5 ± 14.0 | 29.3 ± 15.6 | 0.5 ± 2.7 |
|  | **U=5403, p=0.07** | **U=5996, p=0.6** | **U=5866, p=0.4** | **U=6101, p=0.7** | **U=5272, p=0.04*** |
| **Recombinant Tissue Plasminogen Activator (rtPA)** |  |  |  |  |  |
| Yes | 37.8 ± 20.0 | 5.7 ± 3.7 | 27.2 ± 13.8 | 28.1 ± 14.0 | 1.2 ± 10.1 |
| No | 37.5 ± 22.7 | 5.6 ± 4.1 | 24.6 ± 15.5 | 31.5 ± 19.1 | 0.8 ± 4.8 |
|  | **U=6082, p=0.9** | **U=5933, p=0.7** | **U=5321, p=0.09** | **U=5804, p=0.5** | **U=5341, p=0.1** |
| **Occlusion Locations** |  |  |  |  |  |
| M1 | 36.6 ± 22.2 | 5.5 ± 3.7 | 25.3 ± 15.5 | 31.2 ± 18.4 | 1.4 ± 9.4 |
| Intracranial ICA/terminus | 34.8 ± 22.3 | 5.9 ± 4.3 | 25.1 ± 15.8 | 33.4 ± 17.5 | 0.7 ± 4.0 |
| M2 | 36.0 ± 22.3 | 6.3 ± 5.3 | 28.8 ± 13.7 | 28.8 ± 16.3 | 0.1 ± 0.2 |
| Basilar | 46.9 ± 18.9 | 4.2 ± 3.0 | 17.0 ± 9.7 | 31.8 ± 19.1 | 0.2 ± 0.6 |
| Cervical ICA | 41.6 ± 28.8 | 6.6 ± 5.5 | 23.9 ± 18.4 | 27.8 ± 22.6 | 0.1 ± 0.3 |
| Vertebral | 30.4 ± 32.4 | 5.1 ± 5.7 | 38.6 ± 29.1 | 25.9 ± 10.4 | 0.0 ± 0.0 |
| P2 | 38.0 ± 0.0 | 7.8 ± 0.0 | 33.3 ± 0.0 | 20.9 ± 0.0 | 0.0 ± 0.0 |
| Multiple | 49.9 ± 21.3 | 4.8 ± 2.7 | 22.6 ± 12.1 | 22.6 ± 14.1 | 0.1 ± 0.3 |
|  | **H(7)=12.7, p=0.08** | **H(7)=3.0, p=0.9** | **H(7)=8.1, p=0.3** | **H(7)=11.2, p=0.1** | **H(7)=1.9, p=0.9** |
| **Suspected Etiology** |  |  |  |  |  |
| Large-artery atherosclerosis | 44.8 ± 23.0 | 4.9 ± 3.5 | 21.7 ± 10.5 | 28.3 ± 18.0 | 0.3 ± 1.5 |
| Cardioembolism | 37.2 ± 21.1 | 5.8 ± 3.9 | 26.3 ± 15.5 | 29.8 ± 15.8 | 0.9 ± 7.7 |
| Cryptogenic | 38.7 ± 22.0 | 4.8 ± 3.1 | 24.1 ± 15.3 | 29.9 ± 19.5 | 2.6 ± 11.0 |
| Other | 28.1 ± 22.4 | 6.7 ± 5.2 | 27.1 ± 16.1 | 37.8 ± 25.2 | 0.3 ± 0.8 |
|  | **H(3)=7.5, p=0.06** | **H(3)=3.2, p=0.4** | **H(3)=1.9, p=0.6** | **H(3)=2.4, p=0.5** | **H(3)=0.5, p=0.9** |
| **NIH Stroke Scale/Score (NIHSS)** |  |  |  |  |  |
| Mild Stroke <6 | 35.1 ± 29.8 | 5.5 ± 4.7 | 27.7 ± 15.6 | 28.7 ± 18.2 | 3.0 ± 8.3 |
| Moderate Stroke 6-15 | 41.8 ± 21.4 | 4.9 ± 3.2 | 24.1 ± 13.9 | 29.0 ± 16.1 | 0.1 ± 0.1 |
| Severe Stroke >15 | 35.6 ± 21.1 | 6.0 ± 4.1 | 26.0 ± 15.4 | 31.1 ± 18.2 | 1.2 ± 8.7 |
|  | **H(2)=3.5, p=0.2** | **H(2)=3.1, p=0.2** | **H(2)=0.8, p=0.7** | **H(2)=0.4, p=0.8** | **H(2)=1.7, p=0.4** |
| **Discharged NHISS** |  |  |  |  |  |
| Mild Stroke <6 | 40.1 ± 22.4 | 5.4 ± 4.0 | 26.1 ± 15.4 | 28.0 ± 14.9 | 0.4 ± 2.6 |
| Moderate Stroke 6-15 | 39.2 ± 20.9 | 5.6 ± 3.3 | 23.3 ± 14.1 | 31.8 ± 18.1 | 0.1 ± 0.4 |
| Severe Stroke >15 | 32.4 ± 21.9 | 6.1 ± 4.7 | 24.7 ± 14.0 | 33.1 ± 22.3 | 3.7 ± 15.9 |
|  | **H(2)=3.5, p=0.2** | **H(2)=1.1, p=0.6** | **H(2)=1.4, p=0.5** | **H(2)=1.6, p=0.5** | **H(2)=0.4, p=0.8** |
| **Modified Thrombolysis in Cerebral Infarction**  **(mTICI) score** |  |  |  |  |  |
| 0 | 20.8 ± 17.8 | 3.3 ± 2.1 | 42.3 ± 2.6 | 33.4 ± 17.5 | 0.1 ± 0.2 |
| 1 | 22.2 ± 18.3 | 9.0 ± 7.8 | 5.8 ± 1.1 | 62.7 ± 12.0 | 0.3 ± 0.5 |
| 2a | 39.6 ± 30.4 | 4.6 ± 1.7 | 34.5 ± 26.6 | 21.2 ± 11.2 | 0.1 ± 0.0 |
| 2b | 31.1 ± 22.7 | 5.6 ± 4.5 | 28.0 ± 15.3 | 35.0 ± 23.0 | 0.4 ± 0.9 |
| 2c | 39.7 ± 23.2 | 5.3 ± 3.9 | 23.6 ± 12.5 | 30.2 ± 16.4 | 1.2 ± 6.9 |
| 3 | 39.0 ± 20.6 | 5.8 ± 3.8 | 25.5 ± 15.4 | 28.7 ± 15.9 | 1.1 ± 8.3 |
|  | **H(5)=6.2, p=0.3** | **H(5)=2.5, p=0.8** | **H(5)=9.9, p=0.08** | **H(5)=7.2, p=0.2** | **H(5)=1.5, p=0.9** |
| *p<0.05, **p<0.01 Dunn's multiple comparisons test vs ^a^ ≥90 years age | | | | | |

**Supplementary Table 2.** Comparison graphs of histologic composition percentages in patients with single and multiple risk factors.

| **CHARACTERISTIC** | | **SAMPLES** | | **HISTOLOGICAL COMPOSITION (MSB) Mean ± SD** | | | | |
| --- | --- | --- | --- | --- | --- | --- | --- | --- |
| **Single Risk Factor** | | **n** | **(%)** | **RBC** | **WBC** | **Fibrin** | **Platelets/other** | **Collagen** |
| Hypertension | | 18 | 24.3 | 39.6 ± 24.8 | 4.5 ± 3.8 | 27.5 ± 18.3 | 28.3 ± 17.4 | 0.2 ± 0.5 |
| Atrial fibrillation | | 14 | 18.9 | 40.8 ± 20.0 | 6.6 ± 3.4 | 21.4 ± 12.9 | 31.1 ± 14.9 | 0.1 ± 0.1 |
| Hyperlipidaemia | | 3 | 4.1 | 33.7 ± 6.1 | 5.5 ± 3.9 | 34.7 ± 2.4 | 26.0 ± 11.2 | 0.0 ± 0.0 |
| Coronary artery disease | | 2 | 2.7 | 38.4 ± 6.4 | 4.4 ± 0.9 | 28.2 ± 7.6 | 29.0 ± 13.1 | 0.1 ± 0.0 |
| Smoking | | 10 | 13.5 | 46.4 ± 22.9 | 6.6 ± 5.0 | 22.8 ± 14.7 | 24.0 ± 9.3 | 0.1 ± 0.2 |
| Diabetes mellitus | | 6 | 8.1 | 32.3 ± 17.1 | 8.9 ± 7.3 | 20.6 ± 5.9 | 37.6 ± 21.6 | 0.7 ± 1.4 |
| Other | | 12 | 16.2 | 44.2 ± 25.1 | 5.2 ± 3.4 | 18.5 ± 9.4 | 32.1 ± 29.4 | 0.1 ± 0.2 |
| None | | 9 | 12.2 | 46.3 ± 19.3 | 6.4 ± 4.3 | 23.1 ± 14.3 | 24.0 ± 10.0 | 0.3 ± 0.8 |
| **Total** | | **74** | **100** | **H(7)=3.4, p=0.9** | **H(7)=6.5, p=0.5** | **H(7)=6.2, p=0.5** | **H(7)=2.6, p=0.9** | **H(7)=12.1, p=0.1** |
|  | | | | | | | | |
| **Multiple Risk Factors** | | **n** | **(%)** | **RBC** | **WBC** | **Fibrin** | **Platelets/other** | **Collagen** |
| Hypertension | | 122 | 26.5 | 36.2 ± 21.5 | 5.4 ± 3.6 | 26.2 ± 15.0 | 30.9 ± 16.9 | 1.2 ± 8.7 |
| Atrial fibrillation | | 86 | 18.7 | 35.5 ± 21.1 | 5.7 ± 3.9 | 26.9 ± 15.2 | 31.0 ± 16.4 | 0.8 ± 5.6 |
| Hyperlipidaemia | | 82 | 17.8 | 35.9 ± 22.2 | 5.4 ± 3.8 | 27.2 ± 16.2 | 30.8 ± 16.9 | 0.6 ± 3.1 |
| Coronary artery disease | | 48 | 10.4 | 32.4 ± 20.1 | 5.3 ± 3.6 | 29.7 ± 17.5 | 30.4 ± 14.1 | 2.2 ± 13.3 |
| Smoking | | 37 | 8.0 | 33.2 ± 20.6 | 5.7 ± 4.3 | 26.8 ± 14.7 | 33.9 ± 17.8 | 0.4 ± 1.4 |
| Diabetes mellitus | | 33 | 7.2 | 31.3 ± 17.8 | 6.4 ± 4.1 | 28.8 ± 15.4 | 30.6 ± 17.3 | 3.0 ± 16.0 |
| Other | | 51 | 11.1 | 31.7 ± 24.4 | 5.4 ± 3.4 | 24.6 ± 15.4 | 35.3 ± 20.5 | 3.1 ± 14.6 |
| None | | 1 | 0.2 | 4.7 ± 0.0 | 7.9 ± 0.0 | 38.5 ± 0.0 | 47.9 ± 0.0 | 1.1 ± 0.0 |
| **Total** | | **460** | **100** | **H(7)=6.4, p=0.5** | **H(7)=2.9, p=0.9** | **H(7)=4.0, p=0.8** | **H(7)=4.8, p=0.7** | **H(7)=10.0, p=0.2** |
|  | | | | | | | | |
| **Number of risk factors** | **0** | **1** | **2** | **3** | **4** | **5** | **6** | **Total** |
| n (patients) | 9 | 65 | 74 | 42 | 22 | 16 | 3 | 231 |

**Supplementary Figure 1.** Histopathological appearance of a representative MSB-stained clot. Clot sample scanned at 40x magnification. General appearance (A), arrows indicate RBC (yellow) (B), WBC (purple) (C), fibrin (red) (D), platelets/other (grey pink) (E), and collagen (blue) (F).

**
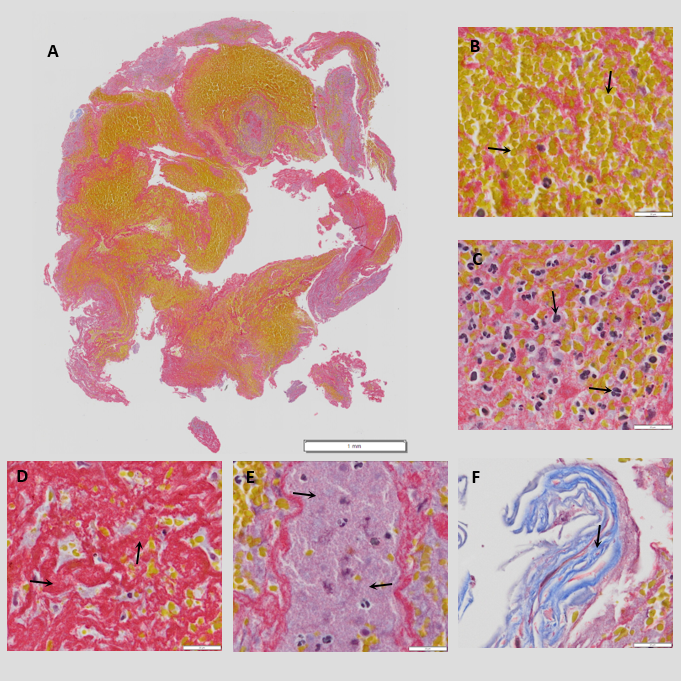
**

**Supplementary Figure 2.** Bland-Altman Plot (Bias = 1.1% (Standard Deviation (SD) ± 22.2,

95% confidence interval limits of agreement ranged from −42.4 to 44.5%)


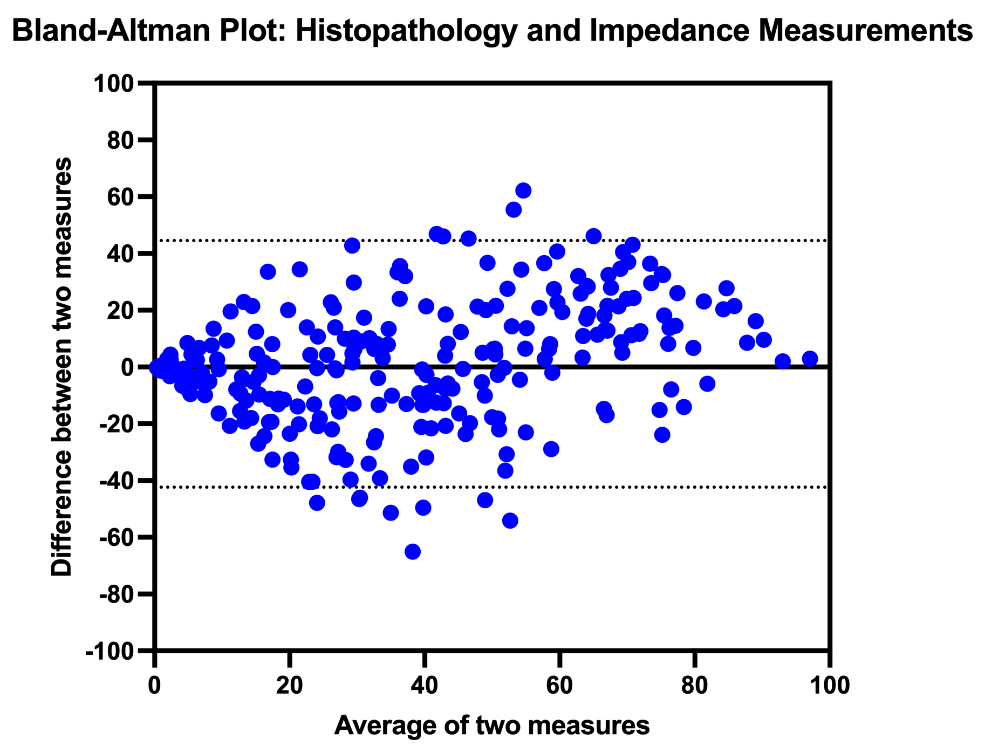

Supplement: Supplementary Material [file mmc1.docx]
